# 3′-UTR Polymorphisms of *MTHFR* and *TS* Associated with Osteoporotic Vertebral Compression Fracture Susceptibility in Postmenopausal Women

**DOI:** 10.3390/ijms19030824

**Published:** 2018-03-12

**Authors:** Tae-Keun Ahn, Jung Oh Kim, Hyun Woo Kim, Han Sung Park, Jeong Hyun Shim, Alexander E. Ropper, In Bo Han, Nam Keun Kim

**Affiliations:** 1 Department of Orthopedics, CHA Bundang Medical Center, CHA University, Seongnam 13496, Korea; ajh329@gmail.com; 2 Department of Biomedical Science, College of Life Science, CHA University, Seongnam 13488, Korea; jokim8505@gmail.com (J.O.K.); woowoo17@naver.com (H.W.K.); hahnsung@naver.com (H.S.P.); 3 Department of Neurosurgery, Shim Jeong Hospital, Seoul 08761, Korea; gumjung70@gmail.com; 4 Department of Neurosurgery, Baylor Colleage of Medicine, Houston, TX 77030, USA; aropper@gmail.com; 5 Department of Neurosurgery, CHA Bundang Medical Center, CHA University, Seongnam 13496, Korea

**Keywords:** methylenetetrahydrofolate reductase, thymidylate synthase, polymorphism, osteoporosis, compression fracture

## Abstract

Postmenopausal osteoporosis is one of the most prominent diseases in postmenopausal women and it is increasing in prevalence with the aging population. Furthermore, osteoporosis and osteoporotic vertebral compression fractures (OVCFs) are related to mortality and decreased quality of life. Therefore, searching for biomarkers that are able to identify postmenopausal women who are at high risk of developing OVCFs is an effective strategy for improving the quality of life of patients and alleviating social and economic burdens. In this study, we investigated methylenetetrahydrofolate reductase (*MTHFR*) and thymidylate synthase (*TS*) gene polymorphisms in postmenopausal women with OVCF. We recruited 301 postmenopausal women and performed genotyping for the presence of *MTHFR* 2572C>A, 4869C>G and TS 1100C>T, 1170A>G. Genotyping was analyzed using the polymerization chain reaction restriction fragment length polymorphism assay. MTHFR 2572C>A and TS 1100C>T were associated with the prevalence of osteoporosis (MTHFR 2572CC versus CA+AA: odd ratio [OR] adjusted age, hypertention [HTN], and diabetes mellitus [DM] = 0.49, *p* = 0.012) and the occurrence of OVCFs (MTHFR 2572CC versus CA+AA: OR adjusted age, HTN, and DM = 0.38, *p* = 0.013; TS 1100CC versus CT+TT: OR adjusted age, HTN, and DM = 0.46, *p* = 0.02). Our novel finding is the identification of MTHFR and TS genetic variants that decrease susceptibility to OVCFs. Our findings suggest that polymorphisms in the *MTHFR* and *TS* genes are associated with susceptibility to osteoporosis and OVCFs in postmenopausal women.

## 1. Introduction

Osteoporosis is a systemic skeletal disorder characterized by low bone mass and a deterioration of bone structure, both of which are associated with bone fragility and increased fracture risk [1]. Previous studies have presented various factors associated with the occurrence of osteoporosis, including aging, obesity, prior fractures, smoking status, metabolic disorders of vitamin B12, genetic variants, and postmenopausal estrogen deficiency [2]. Postmenopausal osteoporosis is associated with mortality, decreased quality of life, and fractures [3]. Menopause elicits changes in homeostatic regulation as indicated by reduced reproductive hormone levels, abnormal metabolism, and increased total homocysteine (tHcy) levels, all of which are related to an elevated occurrence of osteoporosis [4,5].

Hcy is an amino acid that is not obtained from the diet and is not used in protein synthesis. Hcy is biosynthesized from methionine and is subsequently either recycled back into methionine or converted into cysteine by B-group vitamins [6]. An elevated plasma concentration of tHcy has been associated with an increased risk of osteoporotic fractures [7,8,9]. Some in vivo studies have reported that Hcy stimulates osteoclast differentiation and induces apoptosis of osteoblast-lineage cells [10,11].

Osteoporosis is associated with various susceptible genes. The methylenetetrahydrofolate reductase (MTHFR) gene and thymidylate synthase (TS) gene are involved in tHcy/folate metabolism, affect plasma concentrations of tHcy, and have been known as candidates that may cause osteoporosis [12]. MTHFR, which catalyzes the conversion of 5,10-methylenetetrahydrofolate (5,10-MTHF) to 5-methyltetrahydrofolate (5-MTHF), plays a pivotal role in supplying the methyl group for Hcy in Hcy/folate metabolism. Two single nucleotide polymorphisms (SNPs) of the MTHFR gene, MTHFR 677C>T and 1298A>C, have been well studied and are known to be associated with various diseases such as cancer, coronary heart disease, neural tube defect, ischemic stroke, and osteoporotic fractures [13,14,15,16,17,18]. TS catalyzes the conversion of deoxyuridine monophosphate (dUMP) to deoxythymidine monophosphate (dTMP) by the transfer of a 5,10-MTHF methyl group. Polymorphisms in the TS gene lead to an accumulation of Hcy, a decrease in methionine synthesis, and a subsequent decrease in TS levels due to a defect in dTMP synthesis. Several articles have described that polymorphisms of the TS gene increase the risk of breast cancer, colon cancer, gastric cancer, and non-Hodgkin’s lymphoma. The risk of these diseases has been associated with two common polymorphisms of the TS gene: a variable number of tandem repeats in the TS enhance region of the 5′-untranslated region (UTR), and a 6-bp insertion/deletion in the 3′-UTR [19,20,21,22]. Several lines of evidence have revealed that SNPs in the 3′-UTR can alter gene expression by microRNAs (miRNAs), which attenuate or degrade the transcription of the target messenger RNAs. An association between SNPs in the 3′-UTR and some diseases, including cancer and cardiovascular disease, has been previously suggested [23,24].

Many previous studies have reported an association between osteoporosis and genetic variants using genome-wide association studies and candidate gene association studies. Especially, there are many reports of SNPs associated with genes predicted to be involved in osteoporosis prevalence [25,26,27,28,29,30,31,32,33,34]. However, most genetic variants are related to inflammation [25,26,27,28], extracellular matrix mechanisms [29,30], and vitamin D metabolism [31,32,33,34]. On the other hand, studies on the relationship between vitamin B and gene mutations are still insufficient. Among them, studies on the *MTHFR* gene only include 677C>T SNP studies [32,33,34] and *TS* genes have not been studied. Therefore, we hypothesized that there is an association between osteoporotic vertebral compression fractures (OVCFs) and MTHFR and TS gene polymorphisms in the 3′-UTR.

In our study, four SNPs in MTHFR and TS 3′-UTR were identified by a database search: MTHFR 2572C>A (rs4846049) and 4869C>G (rs1537514), and TS 1100T>C (rs699517) and 1170A>G (rs2790). The minor allele frequencies of four SNPs were all >5% in the Asian population. Little is known about the associations between MTHFR and TS polymorphisms with osteoporosis and OVCF. Therefore, we investigated the genetic association between OVCF and the SNPs of *MTHFR* (rs4846049 and rs1537514) and *TS* (rs699517 and rs2790) genes in Korean postmenopausal women.

## 2. Results

### 2.1. Patient Characteristics

The baseline characteristics of the study subjects are shown in Table 1. The mean ages in the control group and osteoporosis group were 69.36 ± 6.26 years and 69.38 ± 7.25 years, respectively. Of the 143 subjects with osteoporosis, 74 exhibited vertebral compression fractures. When comparing the control group with the osteoporosis and OVCF groups, we found that the patients with osteoporosis and OVCF were significantly more likely to have high blood glucose, decreased folate levels, and low body mass index (BMI). These significant differences were more distinct in patients with OVCF than those with osteoporosis (Table 1).

### 2.2. Genotype Frequencies of MTHFR and TS 3′-UTR Variants

We investigated the genotype distributions for MTHFR and TS 3′-UTR polymorphisms. The genotype frequencies of controls and patients were consistent with expectations under the Hardy–Weinberg equilibrium (HWE). When compared with the control group, MTHFR 2572CA+AA was significantly associated with the decreased prevalence of osteoporosis (CC versus CA+AA: OR adjusted age, HTN, and DM = 0.49, 95% Confidence interval [CI] = 0.29–0.86, *p* = 0.012) and OVCF (CC versus CA+AA: OR adjusted age, HTN, and DM = 0.38, 95% CI = 0.18–0.81, *p* = 0.013). TS 1100CT+TT was also associated with the decreased prevalence of OVCF (CC versus CT+TT: OR adjusted age, HTN, and DM = 0.46, 95% CI = 0.24–0.89, *p* = 0.02). For TS 1170A>G, regardless of dominant or recessive genotype, both AG and GG genotypes significantly reduced the occurrence of not only osteoporosis but also OVCF (Table 2).

### 2.3. Stratified Analysis for MTHFR and TS 3′-UTR Variants According to Clinical Parameters

To determine whether the alleles were associated with osteoporosis and OVCF prevalence in specific subsets of patients, we conducted a stratified analysis of the data according to age, hypertension, diabetes mellitus (DM), serum folate, and tHcy levels. To establish cut-off values in the ranges of folate and tHcy serum levels, we selected the upper and lower 15% cut-offs for folate and tHcy, respectively. These values correspond to 4.59 nmol/L for folate and 12.68 μmol/L for tHcy (Table 3). For MTHFR 2572CA+AA genotype, there were subset-specific associations with ≥69 years, folate ≤ 4.59 nmol/L, tHcy < 12.68 μmol/L, and non-hypertensive subgroups. There were no subset-specific associations with MTHFR 4869CG+GG and TS 1100AG+GG genotypes. The polymorphisms of TS 1170 gene showed significant associations with all subgroups, showing decreased osteoporosis occurrence regardless of subset settings (Table 3).

### 2.4. Haplotype Analysis

We conducted an allele-based multifactor dimensionality reduction analysis (Table 4). When osteoporosis patients were compared with controls, MTHFR 2572A-4869G was significantly associated with a decreased prevalence of osteoporosis (*p* = 0.032). For TS gene allele combination, TS 1100T-1170A was significantly associated with an increased occurrence of osteoporosis (*p* = 0.04). However, TS 1100T-1170G showed a significant association with a decreased occurrence of osteoporosis (*p* = 0.011) and OVCF (*p* = 0.003) (Table 4).

## 3. Discussion

Osteoporosis is a multifactorial disease resulting from genetic and environmental factors. Family and twin studies have shown high heritability of bone mineral density (BMD), bone geometry, bone turnover, and fracture risk [35,36]. In this respect, many candidate gene studies have found dozens of genes associated with osteoporosis and osteoporotic fractures [35,37,38,39,40]. In addition, epigenetic studies indicate that certain miRNAs also increase the prevalence of osteoporosis and osteoporotic fractures [41,42].

miRNAs are small noncoding RNA molecules containing 21–23 nucleotides that regulate post-transcriptional gene expression by binding to the 3′-UTRs of the target messenger RNA (mRNA). The binding affinity may be affected by SNPs existing in the miRNA target sites. Therefore, polymorphisms in the 3′-UTRs of mRNAs can potentially alter the affinity of the miRNA for the target mRNA. This altered affinity can modulate protein expression by altering their ability to inhibit mRNA translation and promote mRNA degradation [43,44,45].

Among many candidate genes associated with osteoporosis, we are interested in the genes associated with Hcy/folate metabolism and have previously published an article about polymorphisms in the *MTHFR* and *TS* genes related to OVCF. In the present study, we focused on the polymorphisms in the 3′-UTR of the *MTHFR* and *TS* genes. To our knowledge, this is the first study to provide evidence that 3′-UTR polymorphisms of *MTHFR* and *TS* are associated with the risk of osteoporosis and OVCF [12,14].

Our results demonstrate that patients with osteoporosis or OVCF present with high blood glucose, decreased folate levels, and low BMI. These findings are in accordance with previous results in osteoporosis studies. Osteoporosis and risk of osteoporotic fractures are significantly associated with both type 1 and type 2 diabetes [46,47,48,49]. It has been reported that low folate and high tHcy levels in serum have a significant association with osteoporotic fractures [50]. Low BMI is also an important risk factor for low bone mass and increased risk of osteoporotic fractures [51,52]. Although the tHcy level did not show a significant association with osteoporosis and OVCF in this study, there was a trend in that the serum tHcy level was higher in the osteoporosis and OVCF groups than the control group.

Since linkage studies identified an association between low BMD and the 1p36 region on chromosome 1 in the vicinity of the *MTHFR* gene, many studies have focused on the polymorphism *MTHFR* 677C>T. However, these studies have reported inconsistent results in the association between osteoporosis and *MTHFR* genotypes [8,14,53,54,55,56]. Unlike previous studies, we investigated the polymorphisms in the 3′-UTR of the *MTHFR* gene. Our results show that *MTHFR* 2572CA+AA is significantly associated with a decreased risk of osteoporosis and OVCF and that the allele combination of *MTHFR* 2572A-4869G is significantly associated with a decreased risk of osteoporosis. Several previous studies have described that the polymorphism of the *MTHFR* gene results in hyperhomocysteinemia, a state in which osteoporosis may occur [13,57]. However, our study shows contradictory results whereby the polymorphism in the 3′-UTR of the *MTHFR* gene reduced the risk of osteoporosis and OVCF. Wu et al. [13] described that the polymorphism of *MTHFR*, 2572C>A, is significantly associated with increased risk for coronary heart disease and is regulated by miR-149. Ahn and Kim et al. reported that the polymorphism of miR-149 is significantly associated with decreased risk for osteoporosis and OVCF. However, the protective role of the polymorphism of *MTHFR* 2572C>A should be interpreted with caution, as we did not demonstrate in vitro functional effects of the genetic variation and did not establish a direct interaction of the target gene with the miRNA.

TS is an enzyme involved in Hcy/folate metabolism and the *TS* gene is known as a candidate gene for osteoporosis. However, only one article has studied the association between the polymorphism of the *TS* gene and the risk of osteoporosis and osteoporotic fractures [45]. A study about the association between polymorphisms of the 3′-UTR in the *TS* gene and osteoporosis has never been reported. In the present study, *TS* 1100CT+TT was also associated with a decreased risk of OVCF, and the G allele of *TS* 1170A>G was associated with decreased prevalence of both osteoporosis and OVCF. This significant effect was not affected by the subset setting in the stratified analysis. Considering this result, at least in this study population, the G allele may be regarded as a strong factor for decreasing the occurrence of osteoporosis and OVCF.

There are several limitations of this study. The study included only Korean postmenopausal women, and our results cannot be generalized to other racial/ethnic groups because SNPs and allelic combination types vary among ethnic groups. Thus, the present results should be replicated and validated in other ethnic groups and in men. Second, this was a hospital-based case–control study, and the sample size was relatively small. Third, we could not conclusively exclude other potential confounding variables, such as exposure to different environmental factors (e.g., smoking, nutrition, and the amount of calcium and vitamin intake), and this study was not a genome-wide association study; therefore, the additional genetic risk factors affecting osteoporosis and OVCF are unclear. Fourth, we did not identify the miRNAs of the target genes and did not perform in vitro studies supporting this clinical data.

## 4. Materials and Methods

### 4.1. Study Design

This was a case-control study to assess the influence of four SNPs in *MTHFR* 2572C>A (rs4846049), *MTHFR* 4869C>G (rs1537514), *TS* 1100C>T (rs699517), and *TS* 1170A>G (rs699517) on OVCF prevalence in Korean menopausal women. The study was performed in accordance with the principles of the Declaration of Helsinki and the Institutional Review Board of CHA Bundang Medical Center (IRB number: BD2015-043, approval of this research granted in 2 March 2014). All study subjects provided written informed consent to participate in the study and gave signed informed consents for the publication of the images of the patient.

### 4.2. Study Population

The study group included 301 postmenopausal Korean women from the South Korean province of Gyeonggi-do. Postmenopausal women were recruited from the neurosurgery and orthopedic surgery departments at the CHA Bundang Medical Center. We included women who were at least 50 years of age and had not menstruated for at least one year. The diagnosis of osteoporosis was based on the World Health Organization (WHO) criteria (Dual X-ray Absorptiometry (DXA) hip or lumbar BMD T-score ≤ 2.5 standard deviations (SD)) and OVCF was diagnosed when a progressive or newly generated compression fracture was identified after low-energy trauma. The study population consisted of 143 postmenopausal women with osteoporosis and 158 healthy women without osteoporosis (control group). The subjects with osteoporosis consisted of 74 postmenopausal women with OVCF (OVCF group) and 69 postmenopausal women without fractures (non-OVCF group).

All patients with osteoporosis met these eligibility criteria: (1) absence of metabolic diseases such as diffuse idiopathic skeletal hyperostosis, pituitary diseases, hyperthyroidism, rheumatoid arthritis, or hyperparathyroidism; (2) without treatments associated with bone metabolism or blood coagulation, including anticoagulants, oral contraceptives, hormone replacement therapy, glucocorticoids, calcium, or vitamin D; (3) no prior cancer diagnosis; and (4) of Korean descent. All patients were examined using conventional X-radiography, DXA, and magnetic resonance imaging (MRI) to evaluate the fracture configuration and acuity of the fracture. Whole-body bone scanning was used in cases where MRI was contraindicated.

Control subjects were recruited from individuals who visited our hospital for health examinations. The control subjects had BMD T-scores (lumbar spine and hip) higher than −1.0 SD and had no spine or hip fractures on conventional radiography. The exclusion criteria were the same as those used in the patient group. Hypertension and diabetes mellitus (DM) were not considered exclusion criteria and were observed in the subject demographics. Hypertension was defined as a systolic blood pressure (SBP) of 140 mmHg or more, a diastolic blood pressure (DBP) of 90 mm Hg or more, or taking antihypertensive medication. DM was defined when a subject’s fasting plasma glucose level was higher than 126 mg/dL, including patients taking anti-diabetic drugs. Blood was sampled from all the patients for measurement of folate (reference range 3.45–13.77 ng/mL), glucose, homocysteine (reference range < 12 µmol/L), high-density lipoprotein (HDL, reference range > 40 mg/dL), low-density lipoprotein (LDL, reference range < 130 mg/dL), and triglycerides (TG, reference range < 200 mg/dL).

### 4.3. Genetic Analyses

DNA was extracted from leukocytes using the G-DEX II Genomic DNA Extraction kit (Intron Biotechnology, Seongnam, South Korea), according to the manufacturer’s instructions. Genotyping of *MTHFR* 2572C>A, *MTHFR* 4869C>G, *TS* 1100T>C, and *TS* 1170A>G were determined by polymerase chain reaction-restriction fragment length polymorphism (PCR-RFLP).

*MTHFR* 2572C>A was amplified by forward (5′-TTG CCA ACT AAG CCC TCG AAA CAA-3′) and reverse (5′-TGC CAC ATC TCT TCT ACG ATG CCA-3′) primers. The 140-bp PCR product was then digested with 5U *Sty*I. A digestion product of 140-bp represented the AA genotype; fragments of 140-bp and 70-bp represented the CA genotype; and 70-bp products represented the CC genotype. *MTHFR* 4869C>G was amplified by using forward (5′-AGG CAA GCC CCT CAG CCC TT-3′) and reverse (5′-TCC AGC CCT GAG CCC AGA GTC T-3′) primers. The 126-bp product was digested with 3U *Bsm*AI for 16 h at 55 °C. A restriction fragment of 126-bp represented the CC genotype; fragments of 126-bp, 98-bp, and 28-bp represented the CG genotype; and 98-bp and 28-bp products represented the GG genotype. To detect the *TS* 1100T>C and 1170A>G genotypes, PCR amplification was performed with forward (5′-GGT ACA ATC CGC ATC CAA CTA TTA-3′) and reverse (5′-CTG ATA GGT CAC GGA CAG ATT T-3′) primers. The length of the amplified fragment was 170-bp. PCR products were digested with 5U *Ban*II (*TS* 1100T>C) or 3U *Mbo*II (*TS* 1170A>G) for 16 h at 37 °C. For 1100T>C, restriction products of 170-bp identified the TT genotype; products of 170-bp, 108-bp, and 62-bp represented the TC genotype; and 108-bp and 62-bp products represented the CC genotype. For 1170A>G, restriction products of 170-bp identified the AA genotype; products of 170-bp, 142-bp, and 28-bp represented the AG genotype; and 142-bp and 28-bp products represented the GG genotype. To verify the accuracy of the experiments, we randomly selected 30% of the PCR-RFLP samples and confirmed them by Sanger sequencing using the ABI 3730xl DNA Analyzer (Applied Biosystems, Foster City, CA, USA). The concordance of the two methods was 100%.

### 4.4. Statistical Analyses

To analyze baseline characteristics, chi-squared tests were used for categorical data and Student’s *t*-tests for continuous data to compare patient and control baseline data. The genotype and allele combination frequencies were compared between cases (patients with osteoporosis, non-OVCF, and OVCF group) and controls using logistic regression in multivariable-adjusted ORs and Fisher’s exact test in compared simple genotype and allele frequencies. To estimate the relative risk of the various genotypes for patients with osteoporosis in the non-OVCF and OVCF groups, the odds ratio (OR) and 95% confidence interval (CI) were calculated. A probability (*p*) value of 0.05 was considered to indicate statistical significance. ORs were adjusted by possible confounders, including age, hypertension, and DM [58]. These parameters were selected as adjustment variables because they are directly or potentially associated with OVCF. To evaluate the association data by the Benjamini–Hochberg method, we calculated FDR-corrected P values according to the number of genetic markers and stratified groups [59]. The statistical result with unadjusted variable was also demonstrated (Supplementary Table S1) for reference. We checked the statistical normality of the continuous variables for SBP, DBP, FBS, tHcy, folate, BMI, HDL, LDL, TG, and BMD by confirming that they matched the normal distribution through the Kolmogorov–Smirnov test. Among them, tHcy, folate, and TG did not match the normal distribution and a Mann–Whitney test was performed. Statistical analyses were performed in GraphPad Prism 4.0 software (GraphPad Software, Inc., San Diego, CA, USA) and StatsDirect version 2.4.4 software (StatsDirect Ltd., Altrincham, UK), or HAPSTAT 3.0 (University of North Carolina, Chapel Hill, NC, USA). A power analysis on the sample size in the current study was also performed (Supplementary Table S2).

## 5. Conclusions

Our results suggest that the polymorphisms of *MTHFR* 2572C>A, *TS* 1100C>T, and *TS* 1170 A>G are significantly associated with a decreased risk of osteoporosis and/or OVCF. Our results suggest that the polymorphisms in the 3′-UTR of the *MTHFR* and *TS* genes have a significant association with osteoporosis and OVCF. Although these findings have a small impact on the complex pathogenesis for osteoporosis, the data described here could contribute to the pool of SNP variants needed for individual osteoporosis and OVCF risk assessment.

## Figures and Tables

**Table 1:** Baseline characteristics between controls and osteoporosis patients.

Characteristic	Controls (*n* = 158)	Osteoporosis (*n* = 143)	*p* ^1^	OVCF (*n* = 74)	*p* ^2^	Non-OVCF (*n* = 69)	*p* ^3^
Age (years, mean ± SD)	69 ± 6	69 ± 7	0.983	70 ± 9	0.282	71 ± 7	0.207
Hypertension (%)	79	52	0.134	32	0.001	20	<0.0001
SBP (mmHg, mean ± SD)	136 ± 19	127 ± 14	<0.0001	128 ± 15	0.003	127 ± 14	0.0002
DBP (mmHg, mean ± SD)	81 ± 12	76 ± 10	0.0001	77 ± 10	0.012	74 ± 10	0.0001
Diabetes mellitus (%)	22	26	0.392	9	0.050	17	0.645
FBS (mg/dL, mean ± SD)	111.78 ± 28.12	123.56 ± 43.20	0.006	132.56 ± 54.01	0.0002	114.70 ± 26.41	0.466
tHcy (μmol/L, mean ± SD)	9.74 ± 3.04	9.83 ± 4.09	0.830	10.11 ± 3.84	0.441	9.54 ± 4.35	0.690
Folate (nmol/L, mean ± SD)	9.51 ± 6.48	8.28 ± 4.87	0.074	6.57 ± 4.11	0.001	10.27 ± 4.95	0.408
BMI (kg/m^2^, mean ± SD)	24.52 ± 3.11	23.48 ± 3.81	0.045	21.37 ± 6.98	0.005	23.88 ± 2.77	0.173
HDL-chol (mg/dL, mean ± SD)	47.67 ± 12.13	44.78 ± 14.24	0.223	45.16 ± 16.19	0.385	44.42 ± 12.27	0.180
LDL-chol (mg/dL, mean ± SD)	130.30 ± 44.64	107.31 ± 39.55	0.002	120.64 ± 42.64	0.281	95.18 ± 32.38	<0.0001
TG (mg/dL, mean ± SD)	152.85 ± 87.54	146.81 ± 81.90	0.567	159.05 ± 74.81	0.633	133.89 ± 87.61	0.169
BMD (g/cm^2^, mean ± SD)	≥−1.0	−3.04 ± 0.94	-	−2.93 ± 1.21	-	−3.13 ± 0.62	-

OVCF, osteoporotic vertebral compression fracture; SBP, systolic blood pressure; DBP, diastolic blood pressure; FBS, fasting blood sugar; tHcy, total homocysteine; BMI, body mass index; HDL-chol, high density lipoprotein cholesterol; LDL-chol, low density lipoprotein cholesterol; TG, triglyceride; BMD, bone mineral density. ^1^ *p*-value was compared between osteoporosis and control groups; ^2^ *p*-value was compared between OVCF and control groups; ^3^ *p*-value was compared between non-OVCF and control groups.

**Table 2:** Genotype frequencies of methylenetetrahydrofolate reductase (*MTHFR*) and thymidylate synthase (*TS*) gene polymorphisms in osteoporosis.

Genotypes	Control	Case	ORs Adjusted Age, HTN, and DM (95% CI) ^1^	*p* ^2^	*p* ^5^	OVCF	ORs Adjusted Age, HTN, and DM (95% CI) ^1^	*p* ^3^	*p* ^5^	Non-OVCF	ORs Adjusted Age, HTN, and DM (95% CI) ^1^	*p* ^4^	*p* ^5^
	(*n* = 158)	(*n* = 143)				(*n* = 74)				(*n* = 69)			
*MTHFR* 2572C>A													
CC	103 (65.2)	111 (77.6)	1.000 (reference)			62 (83.8)	1.000 (reference)			49 (71.0)	1.000 (reference)		
CA	52 (32.9)	29 (20.3)	0.494 (0.285–0.857)	0.012	0.024	11 (14.9)	0.382 (0.179–0.813)	0.013	0.026	18 (26.1)	0.682 (0.354–1.314)	0.253	0.337
AA	3 (1.9)	3 (2.1)	N/A	N/A		1 (1.4)	N/A	N/A		2 (2.9)	N/A	N/A	
Dominant (CC vs. CA+AA)			0.516 (0.303–0.880)	0.015	0.030		0.397 (0.191–0.825)	0.013	0.026		0.709 (0.375–1.340)	0.29	0.387
Recessive (CC+CA vs. AA)			N/A	N/A			N/A	N/A			N/A	N/A	
HWE-*P*	0.288	0.885											
*MTHFR* 4869C>G													
CC	135 (85.4)	132 (92.3)	1.000 (reference)			68 (91.9)	1.000 (reference)			64 (92.8)	1.000 (reference)		
CG	23 (14.6)	11 (7.7)	0.454 (0.201–1.026)	0.058	0.077	6 (8.1)	0.503 (0.181–1.395)	0.187	0.187	5 (7.2)	0.487 (0.173–1.367)	0.172	0.337
GG	0 (0.0)	0 (0.0)	N/A	N/A		0 (0.0)	N/A	N/A		0 (0.0)	N/A	N/A	
Dominant (CC vs. CG+GG)			0.454 (0.201–1.026)	0.058	0.077		0.503 (0.181–1.395)	0.187	0.187		0.487 (0.173–1.367)	0.172	0.344
Recessive (CC+CG vs. GG)			N/A	N/A			N/A	N/A			N/A	N/A	
HWE-*P*	0.324	0.632											
*TS* 1100C>T													
CC	92 (58.2)	95 (66.4)	1.000 (reference)			56 (75.7)	1.000 (reference)			39 (56.5)	1.000 (reference)		
CT	58 (36.7)	40 (28.0)	0.886 (0.314–2.497)	0.819	0.819	17 (23.0)	0.492 (0.252–0.961)	0.038	0.051	23 (33.3)	0.952 (0.503–1.804)	0.881	0.887
TT	8 (5.1)	8 (5.6)	0.625 (0.215–1.820)	0.389	0.389	1 (1.4)	0.249 (0.030–2.067)	0.198	0.198	7 (10.1)	2.417 (0.784–7.454)	0.125	0.125
Dominant (CC vs. CT+TT)			0.781 (0.283–2.158)	0.634	0.634		0.462 (0.241–0.886)	0.02	0.027		1.114 (0.615–2.021)	0.721	0.721
Recessive (CC+CT vs. TT)			1.355 (0.835–2.200)	0.219	0.219		0.303 (0.037–2.485)	0.266	0.266		2.433 (0.817–7.245)	0.11	0.110
HWE-*P*	0.497	0.280											
*TS* 1170A>G													
AA	64 (40.5)	104 (72.7)	1.000 (reference)			48 (64.9)	1.000 (reference)			56 (81.2)	1.000 (reference)		
AG	76 (48.1)	34 (23.8)	0.276 (0.165–0.460)	<0.0001	0.0004	24 (32.4)	0.413 (0.221–0.774)	0.006	0.024	10 (14.5)	0.147 (0.068–0.320)	<0.0001	0.0004
GG	18 (11.4)	5 (3.5)	0.168 (0.059–0.475)	0.001	0.002	2 (2.7)	0.086 (0.011–0.672)	0.019	0.038	3 (4.3)	0.137 (0.035–0.527)	0.004	0.008
Dominant (AA vs. AG+GG)			0.255 (0.157–0.415)	<0.0001	0.0004		0.354 (0.192–0.653)	0.001	0.004		0.146 (0.072–0.298)	<0.0001	0.0004
Recessive (AA+AG vs. GG)			0.281 (0.101–0.778)	0.015	0.030		0.129 (0.017–0.994)	0.049	0.098		0.275 (0.075–1.007)	0.051	0.102
HWE-*P*	0.520	0.267											

OR, odd ratio; CI, confidence interval; HTN, hypertension; DM, diabetes mellitus; HWE, Hardy–Weinberg equilibrium; N/A, not applicable. ^1^ The odds ratio (OR) adjusted on the basis of risk factors, such as age, hypertension, diabetes mellitus; ^2^ *p*-value was compared between osteoporosis and control groups; ^3^ *p*-value was compared between OVCF and control groups; ^4^ *p*-value was compared between non-OVCF and control groups; ^5^ *p*-value calculated by false discovery rate test.

**Table 3:** Stratified effects of *MTHFR* and *TS* gene polymorphisms on osteoporosis prevalence.

Variables	*MTHFR* 2572 (CC vs. CA+AA)			*MTHFR* 4869 (CC vs. CG+GG)			*TS* 1100 (CC vs. CT+CC)			*TS* 1170 (AA vs. AG+GG)		
	ORs Adjusted Age, HTN, and DM (95% CI) ^1^	*p*	*p* ^4^	ORs Adjusted Age, HTN, and DM (95% CI) ^1^	*p*	*p* ^4^	ORs Adjusted Age, HTN, and DM (95% CI) ^1^	*p*	*p* ^4^	ORs Adjusted Age, HTN, and DM (95% CI) ^1^	*p*	*p* ^4^
Hypertension												
No (*n* = 170)	0.417 (0.204–0.856)	0.017	0.034	0.426 (0.141–1.283)	0.129	0.152	0.620 (0.322–1.193)	0.152	0.152	0.238 (0.122–0.465)	<0.0001	0.0004
Yes (*n* = 131)	0.773 (0.352–1.697)	0.521	0.695	0.649 (0.211–1.997)	0.451	0.695	1.042 (0.504–2.153)	0.912	0.912	0.252 (0.118–0.539)	0.0004	0.002
Diabetes mellitus												
No (*n* = 253)	0.597 (0.337–1.060)	0.078	0.156	0.567 (0.254–1.264)	0.165	0.22	0.724 (0.429–1.224)	0.228	0.228	0.262 (0.151–0.454)	<0.0001	0.0004
Yes (*n* = 48)	0.381 (0.099–1.474)	0.162	0.243	N/A	N/A		1.169 (0.302–4.521)	0.821	0.821	0.142 (0.039–0.522)	0.003	0.009
Folate ^2^												
>4.6 nmol/L (*n* = 244)	0.709 (0.404–1.245)	0.232	0.233	0.554 (0.240–1.280)	0.167	0.233	0.724 (0.426–1.231)	0.233	0.233	0.240 (0.138–0.417)	<0.0001	0.0004
≤4.6 nmol/L (*n* = 57)	0.057 (0.006–0.520)	0.011	0.023	N/A	N/A		1.339 (0.368–4.867)	0.658	0.658	0.204 (0.057–0.734)	0.015	0.023
Total homocysteine ^3^												
≥12.7 μmol/L (*n* = 48)	0.306 (0.063–1.489)	0.143	0.286	1.052 (0.054–20.670)	0.973	0.973	0.852 (0.239–3.034)	0.805	0.973	0.278 (0.066–1.166)	0.08	0.286
<12.7 μmol/L (*n* = 253)	0.599 (0.338–1.062)	0.042	0.084	0.457 (0.193–1.082)	0.075	0.1	0.745 (0.439–1.265)	0.276	0.276	0.263 (0.155–0.446)	<0.0001	0.0004
Age												
≥69	0.467 (0.231–0.945)	0.034	0.068	0.619 (0.210–1.820)	0.383	0.511	0.878 (0.449–1.717)	0.704	0.704	0.131 (0.064–0.269)	<0.0001	0.0004
<69	0.669 (0.303–1.476)	0.319	0.319	0.407 (0.124–1.333)	0.138	0.276	0.646 (0.320–1.305)	0.224	0.299	0.509 (0.259–1.002)	0.051	0.204

OR, odd ratio; CI, confidence interval; HTN, hypertension; DM, diabetes mellitus; HWE, Hardy–Weinberg equilibrium. ^1^ The odds ratio (OR) adjusted on the basis of risk factors, such as age, hypertension, diabetes mellitus; ^2^ Folate 4.59 nmol/L was the lower 15% cut-off folate level in osteoporosis patients and controls; ^3^ Homocysteine 12.68 μmol/L was the upper 15% cut-off homocysteine level in osteoporosis patients and controls; ^4^ *p*-value calculated by the false discovery rate test.

**Table 4:** Comparison of genotype frequencies of *MTHFR* and *TS* gene allele combinations between the osteoporosis and control subjects.

Haplotypes	Control (2*n* = 316)	Case (2*n* = 286)	OR (95% CI) ^1^	*p*	*p* ^2^	OVCF (2*n* = 148)	OR (95% CI) ^1^	*p*	*p* ^2^	Non-OVCF (2*n* = 138)	OR (95% CI) ^1^	*p*	*p* ^2^
*MTHFR* 2572/4869													
C-C	258 (81.7)	216 (87.0)	1.000 (reference)			125 (84.2)	1.000 (reference)			116 (84.2)	1.000 (reference)		
A-C	35 (11.1)	23 (9.2)	0.785 (0.450–1.369)	0.393	0.393	18 (12.3)	1.061 (0.578–1.949)	0.876	0.876	17 (12.3)	1.080 (0.581–2.007)	0.873	0.873
A-G	23 (7.3)	8 (3.1)	0.416 (0.182–0.948)	0.032	0.064	5 (3.5)	0.449 (0.167–1.208)	0.140	0.280	5 (3.5)	0.484 (0.179–1.304)	0.199	0.398
*TS* 1100/1170													
C-A	74 (23.4)	49 (19.6)	1.000 (reference)			34 (22.8)	1.000 (reference)			31 (22.8)	1.000 (reference)		
T-A	130 (41.1)	161 (65.0)	1.870 (1.218–2.872)	0.004	0.008	95 (64.0)	1.578 (0.972–2.562)	0.072	0.072	88 (64.0)	1.604 (0.974–2.641)	0.066	0.066
T-G	112 (35.4)	38 (15.4)	0.512 (0.306–0.858)	0.011	0.032	19 (13.2)	0.373 (0.198–0.702)	0.003	0.006	18 (13.2)	0.387 (0.202–0.742)	0.006	0.012

^1^ Fisher’s exact test; ^2^ *p*-value calculated by the false discovery rate test.

## Supplementary Materials

Supplementary materials can be found at http://www.mdpi.com/1422-0067/19/3/824/s1.
