# Supplementary material for: 3′-UTR Polymorphisms of MTHFR and TS Associated with Osteoporotic Vertebral Compression Fracture Susceptibility in Postmenopausal Women
Source: Int J Mol Sci. 2018 Mar 12;19(3):824. doi: 10.3390/ijms19030824 (PMC5877685; doi:10.3390/ijms19030824)
Supplement: Supplementary file 1 [file ijms-19-00824-s001.pdf]

Supplementary Table 1. Genotype frequencies of *MTHFR* and *TS* genes polymorphisms in osteoporosis by chi-square test.

Genotypes	Control (n=158)	Case (n=143)	OR (95% CI)	*P*[1]	OVCF (n=74)	OR (95% CI)	*P*[2]	non-OVCF (n=69)	OR (95% CI)	*P*[3]
MTHFR 2572C>A										
CC	103 (65.2)	111 (77.6)	1.000 (reference)		62 (83.8)	1.000 (reference)		49 (71.0)	1.000 (reference)	
CA	52 (32.9)	29 (20.3)	0.518 (0.305-0.877)	0.013	11 (14.9)	0.351 (0.171-0.724)	0.003	18 (26.1)	0.728 (0.386-1.373)	0.326
AA	3 (1.9)	3 (2.1)	0.928 (0.183-4.701)	0.928	1 (1.4)	0.554 (0.056-5.441)	0.597	2 (2.9)	1.401 (0.227-8.660)	0.720
Dominant (CC vs CA+AA)			0.540 (0.324-0.901)	0.017		0.363 (0.180-0.730)	0.003		0.764 (0.414-1.413)	0.388
Recessive (CC+CA vs AA)			1.107 (0.220-5.575)	0.902		0.708 (0.072-6.921)	0.760		1.542 (0.252-9.443)	0.645
HWE-*P*	0.288	0.885								
MTHFR 4869C>G										
CC	135 (85.4)	132 (92.3)	1.000 (reference)		68 (91.9)	1.000 (reference)		64 (92.8)	1.000 (reference)	
CG	23 (14.6)	11 (7.7)	0.489 (0.229-1.043)	0.057	6 (8.1)	0.518 (0.201-1.332)	0.152	5 (7.2)	0.459 (0.167-1.261)	0.108
GG	0 (0.0)	0 (0.0)	N/A	N/A	0 (0.0)	N/A	N/A	0 (0.0)	N/A	N/A
Dominant (CC vs CG+GG)			0.489 (0.229-1.043)	0.057		0.518 (0.201-1.332)	0.152		0.459 (0.167-1.261)	0.108
Recessive (CC+CG vs GG)			N/A	N/A		N/A	N/A		N/A	N/A
HWE-*P*	0.324	0.632								
TS 1100C>T										
CC	92 (58.2)	95 (66.4)	1.000 (reference)		56 (75.7)	1.000 (reference)		39 (56.5)	1.000 (reference)	
CT	58 (36.7)	40 (28.0)	0.668 (0.407-1.095)	0.108	17 (23.0)	0.482 (0.255-0.908)	0.024	23 (33.3)	0.936 (0.508-1.724)	0.830
TT	8 (5.1)	8 (5.6)	0.968 (0.349-2.689)	0.951	1 (1.4)	0.205 (0.025-1.686)	0.078	7 (10.1)	2.064 (0.700-6.086)	0.195
Dominant (CC vs CT+TT)			0.704 (0.440-1.126)	0.142		0.448 (0.242-0.831)	0.009		1.072 (0.606-1.899)	0.811
Recessive (CC+CT vs TT)			1.111 (0.406-3.042)	0.838		0.257 (0.032-2.093)	0.136		2.117 (0.736-6.090)	0.171
HWE-*P*	0.497	0.280								
TS 1170A>G										
AA	64 (40.5)	104 (72.7)	1.000 (reference)		48 (64.9)	1.000 (reference)		56 (81.2)	1.000 (reference)	
AG	76 (48.1)	34 (23.8)	0.275 (0.165-0.459)	< 0.0001	24 (32.4)	0.421 (0.233-0.761)	0.004	10 (14.5)	0.150 (0.071-0.319)	< 0.0001
GG	18 (11.4)	5 (3.5)	0.171 (0.061-0.483)	0.000	2 (2.7)	0.148 (0.033-0.669)	0.002	3 (4.3)	0.191 (0.053-0.681)	0.003
Dominant (AA vs AG+GG)			0.255 (0.157-0.415)	< 0.0001		0.369 (0.208-0.654)	0.001		0.158 (0.080-0.313)	< 0.0001
Recessive (AA+AG vs GG)			0.282 (0.102-0.780)	0.008		0.216 (0.049-0.957)	0.016		0.354 (0.101-1.242)	0.073
HWE-P	0.520	0.267								

Abbreviation; HTN, hypertension; DM, diabetes mellitus; HWE, Hardy-Weinberg equilibrium.

[1] *P*-value was compared between osteoporosis and control groups.

[2] *P*-value was compared between OVCF and control groups.

[3] *P*-value was compared between non-OVCF and control groups.

Supplementary Table 2. Statistical powers of genetic associations in the present case-control study

Characteristics	Association	OR adjusted age, HTN, DM (95% CI)	Statistical power (%)
MTHFR 2572CA	Total osteoporosis	0.494 (0.285-0.857)	70.40%
MTHFR 2572 dominant model	Total osteoporosis	0.516 (0.303-0.880)	63.94%
MTHFR 2572CA	OVCF	0.382 (0.179-0.813)	84.80%
MTHFR 2572 dominant model	OVCF	0.397 (0.191-0.825)	68.60%
TS 1100CT	OVCF	0.492 (0.252-0.961)	61.33%
TS 1100 recessive model	OVCF	0.462 (0.241-0.886)	12.45%
TS 1170AG	Total osteoporosis	0.276 (0.165-0.460)	99.93%
TS 1170GG	Total osteoporosis	0.168 (0.059-0.475)	95.25%
TS 1170 dominant model	Total osteoporosis	0.255 (0.157-0.415)	99.99%
TS 1170 recessive model	Total osteoporosis	0.281 (0.101-0.778)	73.32%
TS 1170AG	OVCF	0.413 (0.221-0.774)	80.79%
TS 1170GG	OVCF	0.086 (0.011-0.672)	84.03%
TS 1170 dominant model	OVCF	0.354 (0.192-0.653)	93.48%
TS 1170 recessive model	OVCF	0.129 (0.017-0.994)	61.40%
TS 1170AG	non-OVCF	0.147 (0.068-0.320)	99.99%
TS 1170GG	non-OVCF	0.137 (0.035-0.527)	81.68%
TS 1170 dominant model	non-OVCF	0.146 (0.072-0.298)	99.99%

Abbreviations: OVCF, osteoporotic vetebral compression fracture; HTN, hypertension; DM, diabetes mellitus.
